# Enhanced T Cell Glucose Uptake Is Associated With Progression of Beta-Cell Function in Type 1 Diabetes

**DOI:** 10.3389/fimmu.2022.897047

**Published:** 2022-05-23

**Authors:** Rong Tang, Ting Zhong, Li Fan, Yuting Xie, Juan Li, Xia Li

**Affiliations:** National Clinical Research Center for Metabolic Diseases, Key Laboratory of Diabetes Immunology (Central South University), Ministry of Education, and Department of Metabolism and Endocrinology, The Second Xiangya Hospital of Central South University, Changsha, China

**Keywords:** type 1 diabetes, T cell, cellular glucose uptake, beta-cell function, immunometabolism

## Abstract

**Background:**

Abnormal intracellular glucose/fatty acid metabolism of T cells has tremendous effects on their immuno-modulatory function, which is related to the pathogenesis of autoimmune diseases. However, the association between the status of intracellular metabolism of T cells and type 1 diabetes is unclear. This study aimed to investigate the uptake of glucose and fatty acids in T cells and its relationship with disease progression in type 1 diabetes.

**Methods:**

A total of 86 individuals with type 1 diabetes were recruited to detect the uptake of glucose and fatty acids in T cells. 2-NBDG uptake and expression of glucose transporter 1 (GLUT1); or BODIPY uptake and expression of carnitine palmitoyltransferase 1A(CPT1A) were used to assess the status of glucose or fatty acid uptake in T cells. Patients with type 1 diabetes were followed up every 3-6 months for 36 months, the progression of beta-cell function was assessed using generalized estimating equations, and survival analysis was performed to determine the status of beta-cell function preservation (defined as 2-hour postprandial C-peptide >200 pmol/L).

**Results:**

Patients with type 1 diabetes demonstrated enhanced intracellular glucose uptake of T cells as indicated by higher 2NBDG uptake and GLUT1 expression, while no significant differences in fatty acid uptake were observed. The increased T cells glucose uptake is associated with lower C-peptide and higher hemoglobin A1c levels. Notably, patients with low T cell glucose uptake at onset maintained high levels of C-peptide within 36 months of the disease course [fasting C-petite and 2-hour postprandial C-peptide are 60.6 (95%CI: 21.1-99.8) pmol/L and 146.3 (95%CI: 14.1-278.5) pmol/L higher respectively], And they also have a higher proportion of beta-cell function preservation during this follow-up period (*P*<0.001).

**Conclusions:**

Intracellular glucose uptake of T cells is abnormally enhanced in type 1 diabetes and is associated with beta-cell function and its progression.

## Introduction

Autoreactive T cells have a pivotal pathogenic role in the autoimmune process of beta-cell destruction in type 1 diabetes (T1D), making them ideal targets for immunotherapy strategies aiming to prevent or delay the disease (1, 2). However, clinical evidence witnessed refractory results to standard immunomodulatory and immunosuppressive strategies (3–5). In this sense, the identification and characterization of novel target pathways to control the T cell response to beta cells is still a strong demand (6).

There is a growing appreciation that the function and response of T cells are tightly linked to their bio-energetic metabolism (7). Resting T cells import small amounts of glucose to maintain basal energy and biosynthesis. However, immediately upon activation, T cells strongly upregulate the anabolic process of glycolysis even in the presence of oxygen (Warburg effect) (8, 9). Moreover, it should be noted that the function and metabolism of T cells are bidirectional. On the one hand, T cells with different functions have different intracellular energy metabolism (10); On the other hand, abnormal metabolism of T cells also affects their function (11, 12). In recent years, accumulating evidence demonstrated that abnormal metabolism of T cells is involved in the pathological mechanism of various diseases by regulating immune function (13–15), especially in some autoimmune diseases such as systemic lupus erythematosus and rheumatoid arthritis (16, 17). Further, a few limited animal studies indicated that metabolic inhibition is an effective strategy to weaken the T cell effect in preclinical models of autoimmunity (18–20). Especially, the use of glucose uptake pathway inhibitors can alleviate effectively insulitis in NOD mice (21–23). However, the role of T cell metabolism in T1D patients remains unclear. The possibility of intracellular metabolism of T cells as innovative targets for T1D intervention would be strengthened if the status of intracellular glucose uptake in T cells is well researched. So, we aimed to investigate the intracellular metabolism of T cells and its association with beta-cell function in clinical settings.

## Subjects and Methods

### Subjects and Study Design

A total of 86 patients were recruited from a T1D cohort at the Second Xiangya Hospital, Central South University (ClinicalTrials.gov ID: NCT03610984). Inclusive criteria include 1) diagnosis of diabetes according to the 1999 world health organization (WHO) criteria (24); 2) newly diagnosed with the disease duration within 12 months; 3) positive for at least one of the three islet autoantibodies (glutamic acid decarboxylase antibody [GADA], protein tyrosine phosphatase autoantibody [IA-2A], zinc transporter 8 autoantibody [ZnT8A]; 4) clinically insulin-dependent. Participants who had severe liver/renal impairment, severe heart failure requiring treatment, a history of malignant tumor, acute or chronic infection, or receiving glucocorticoid therapy, pregnancy, and breastfeeding were excluded.

At the time of initial enrollment, demographic information and clinical indicators including gender, age at onset, height, weight, body mass index (BMI), and diabetes autoantibodies, the occurrence of diabetic ketosis/ketoacidosis (DK/DKA) at onset, daily insulin dose, hemoglobin A1c (HbA1c), fasting C-peptide (FCP), 2-hour postprandial C-peptide (2h-CP) by mixed-meal tolerance test (MMTT) were collected and measured for analysis. A standard 543.6 kcal MMTT was performed, with 44.4% of calories coming from carbohydrates, 47.7% from fat, and 7.9% from protein. Long-acting insulin and basal rates (for insulin pump users) were used normally on the day before or on the morning of the study, but their morning dose of short-acting insulin or rapid-acting insulin was withheld as previously reported (25). The MMTT was conducted at least two weeks after the correction of ketoacidosis. After 8 hours of fasting, serum C-peptide levels were tested at 0 and 120 min after a mixed meal. The C-peptide area under curve (AUC-CP) was calculated by the trapezoidal method. Beta-cell function preservation was defined as 2h-CP >200 pmol/L (25).

Participants with T1D were followed up every 3-6 months for a total of 36 months, HbA1c and FCP, 2h-CP by MMTT were measured at each visit. The termination of follow-up was determined when the C-peptide level was <16.5 pmol/L (n=8) or 36 months after onset (n=61). Of all the participants, the mean follow-up time was 28.0 ± 8.8 months, with 8 patients lost to follow-up and 9 patients cut off due to the end of this study.

Normal control (NC) was recruited through a recruitment advertisement at local medical centers. All controls underwent oral glucose tolerance test (OGTT) screening and three islet autoantibodies (GADA, IA-2A, and ZnT8A) testing. Those with normal results were eligible. Also, their medical history excludes those with a positive family history of diabetes, infection, autoimmune disease, severe liver or kidney damage, or steroid hormone therapy. We used the method of group matching, and controls were matched for gender and age with T1D patients.

This study was approved by the Ethics Committee of the Second Xiangya Hospital of Central South University, and all subjects provided written informed consent.

### Assays for HbA1c, C-Peptide, and Islet Autoantibodies

HbA1c was detected by automatic liquid chromatography (VARIANT II hemoglobin test system Bio-Rad Laboratories, Hercules, CA).

C-peptide levels were measured by a chemiluminescence method using the Advia Centaur System kit (Siemens, Munich, Germany). The inter- and intra-assay variation coefficients were 3.7% to 4.1% and 1.0% to 3.3%, respectively, as previously reported (26).

GADA, IA-2A, and ZnT8A were measured by radioimmune assay (RIA) as previously reported (27–29). The positive boundary values of antibodies were: GADA≥0.05 titer, IA-2A≥0.02 titer, ZnT8A≥0.011 titers. The sensitivities were 78%, 74%, and 70%, respectively. The specificity was 96.7%, 96.7%, and 98.9%, respectively. In accordance with the Islet Autoantibody Standardization Program (IASP 2012). The suspicious positive samples were confirmed by secondary testing.

### Flow Cytometry

5x10^5^ – 1x10^6^ cells were harvested and stained for surface and intracellular markers for flow cytometric analysis as described (30). All antibodies were purchased from BD Biosciences or Biolegend. Briefly, living cells were labeled with Fixable Viability Stain 620 dye and then incubated with Fc block for 10 minutes. Surface staining was performed at 4°C using CD4-APC-Cy7, CD8-PE, glucose transporter 1 (GLUT1)-Alexa Fluor 647 in FACS buffer (1% BSA in PBS) for 20 min. Some cells were fixed and permeabilized (BD Biosciences, 554714) for intracellular staining, and mitochondrial intima Carnitine palmitoyltransferase 1A (CPT1A)- Alexa Fluor 647 of these cells were labeled.

To measure glucose uptake or fatty acid uptake, cells were incubated in glucose-free RPMI 1640 medium containing 100 μmol/L 2NBDG (2-[N-(7-nitrobenz-2-oxa-1, 3-diaxOL-4-yl) Amino]-2-deoxyglucose, Thermo Fisher) or in PBS with 20 μmol/L FA-free BSA (A8806; Sigma-Aldrich) containing 2 μmol/L BODIPY FL C16 (4,4-Difluoro-5,7-Dimethyl-4-Bora-​​3a,4a-Diaza-s-Indacene-3-HexadecanoicAcid, Thermo Fisher) for 30 minutes at 37°C in a cell incubator. 2NBDG and BODIPY uptake was quenched by adding 4× volume of ice-cold PBS with 2% FBS, and flow cytometry analysis was performed after surface and intracellular staining.

For all flow cytometry studies, fluorescence was measured using a BD FACSCanto II flow cytometer and data were analyzed with FlowJo 10.0 software. The single fluorescence sample was used to calibrate the instrument regularly. Lymphocytes were circled by the forward/lateral scattering Angle (FSC-A/SSC-A), Adhesion cells were removed by FSC-A/FSC-H. The gating strategy is shown in **Supplement Figure 1**. The percentages of 2NDBG^+^, BODIPY^+^, GLUT1^+^, and CPT1A^+^ cells were marked to assess glucose and fatty acid uptake levels as described in previous publications (31–33).

### Statistical Analysis

SPSS 26.0 and GraphPad Prism 9 were used for analysis. Data are expressed when normally distributed as means ± SD and when abnormally distributed as median (25th, 75th percentiles). Comparison of means using the *T* test or Mann-Whitney *U* test. The enumerative data is expressed as a constituent ratio or rate. Frequency differences were compared using the χ2 test. Spearman or Pearson analysis was used for correlation analysis and Linear regression analysis was applied to the glucose uptake vs clinical characteristics when used as a continuous variable. The analysis of covariance (ANCOVA) was used to adjust for the course of disease. Generalized Estimating Equations (GEE) was used to analyze repeated measurement data during follow-up (25). GEE was employed to compare the C-peptide and HbA1c levels in patients with high and low T cells glucose uptake. Survival analysis of preserved beta-cell function (2h-CP>200 pmol/L) was performed using the Kaplan-Meier method. *P* < 0.05 was considered significant.

## Results

### Basic Characteristics of Subjects

A total of 86 patients (48 males and 38 females) with T1D were recruited. Their median age of onset was 14.4 (11.1, 20.6) years, and the disease course was 3.4 (1.1, 6.4) months. The clinical characteristics of T1D and NC (n=45) individuals were shown in **Table 1**. No significant differences in gender proportion, age, and BMI were found between the two groups.

**Table 1:** The clinical characteristics of T1D patients and NC individuals.

	NC (n = 45)	T1D (n = 86)	*P* value
Male (%)	55.6	55.8	0.977
Age (years)	23.0 (11.0, 25.5)	14.4 (11.1, 20.6)	0.090
Height (cm)	154.2 ± 11.7	155.1 ± 17.3	0.832
Weight (kg)	47.1 ± 17.8	45.8 ± 14.8	0.704
BMI (kg/m^2^)	19.2 ± 4.7	18.4 ± 3.0	0.478
Course of disease (months)	/	3.4 (1.1, 6.4)	
DK/DKA (%)	/	89.3	
FCP (pmol/L)	/	162.9 (112.8, 230.3)	
2h-CP (pmol/L)	/	434.9 (246.7, 725.9)	
HbA1c (%)	/	8.5 ± 2.8	
HbA1c (mmol/mol)		69.4 ± 7.1	
GADA titer	/	0.37 (0.09, 1.04)	
IA-2A titer	/	0.45 (0.00, 1.56)	
ZnT8A titer	/	0.07 (0.00, 0.20)	
Number of positive autoantibodies (%)			
1	/	20.7	
2	/	41.4	
3	/	36.8	
Daily dose of insulin (U/kg)	/	0.49 ± 0.28	

Kolmogorov-Smirnov tested the normal distribution of measurement data, which was expressed as means ± SD, and the abnormal distribution was expressed as the median (25th, 75th percentiles). The t test, Mann-Whitney U test, and χ^2^ test were be used. T1D, type 1diabetes; NC, normal control; BMI, Body mass index; DK/DKA, ketosis/ketoacidosis; FCP, Fasting C-peptide; 2h-CP, 2 hours postprandial C-peptide; HbA1c, hemoglobin A1c; GADA, Glutamic acid decarboxylase antibody; IA-2A, protein tyrosine phosphatase antibody; ZnT8A, Zinc transporter 8 antibody.

### Intracellular T Cell Glucose and Fatty Acid Uptake in T1D

**Figure 1:**
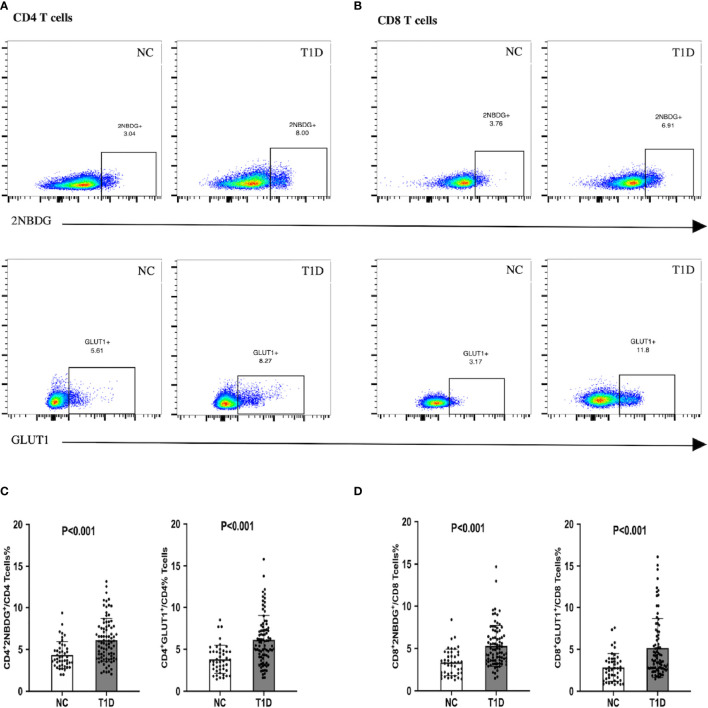
Glucose uptake and GLUT1 expression of CD4+/CD8+ T cells in patients with T1D. T1D patients demonstrated enhanced intracellular glucose uptake. **(A, C)** The proportion of 2NBDG+ and GLUT1+ cells in CD4+ cells. **(B, D)** The proportion of 2NBDG+ and GLUT1+ cells in CD8+ cells. The bar and error bar = mean and SD. T1D, type 1diabetes; NC, normal control; GLUT1, glucose transporter 1.

The fatty acid uptake of CD4^+^ and CD8^+^ T cells was not significantly different between the T1D and NC individuals, as indicated by similar levels of BODIPY and CPT1A (**Supplement Figure 2**). Whereas, the upregulated of 2NBDG and GLUT1 in T cells suggested an enhanced intracellular glucose uptake in T1D patients, compared to the NC group (**Figure 1**).

### Association of T Cells Glucose Uptake With Clinical Features in T1D

**Figure 2:**
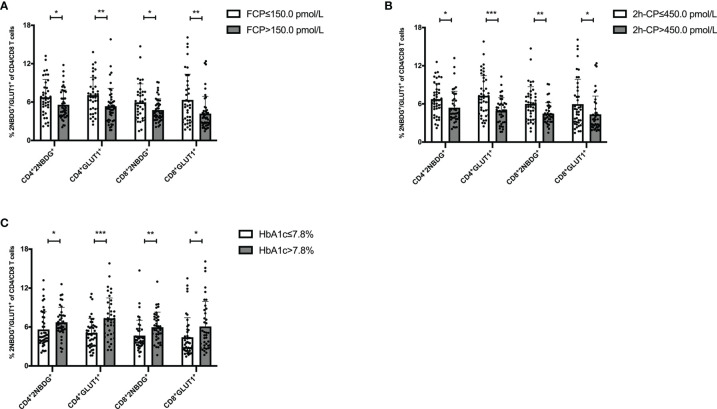
The status of glucose uptake in T cells is related to clinical features of T1D. Patients with FCP>150.0 pmol/L **(A)** 2h-CP >450.0 pmol/L **(B)** and HbA1c ≤ 7.0%/53.0 mmol/mol **(C)** had lower glucose uptake in T cells. The bar and error bar = mean and SD. FCP, Fasting C-peptide; 2h-CP, 2 hours postprandial C-peptide; HbA1c, hemoglobin A1c; 2NBDG, 2-deoxyglucose; GLUT1, glucose transporter 1. *P < 0.05, **P < 0.01, ***P < 0.001.

**Figure 3:**
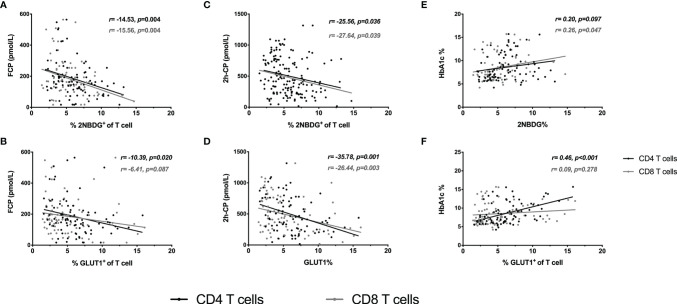
Regression analysis of T cell glucose uptake with clinical characteristics. Linear regression analysis of T-cell glucose uptake vs FCP **(A, B)**, 2h-CP **(C, D)** and HbA1c **(E, F)**. The black line and dots represent CD4+ T cells, the gray line and dots represent CD8+ T cells. FCP, Fasting C-peptide; 2h-CP, 2 hours postprandial C-peptide; HbA1c, hemoglobin A1c; 2NBDG, 2-deoxyglucose; GLUT1, glucose transporter 1.

Further, the association of T cell glucose uptake (measured by 2NBDG uptake and GLUT1 expression) with clinical features in T1D was investigated. Subgroup analysis demonstrated that the patients with better beta-cell function (FCP>150.0 pmol/L or 2h-CP >450.0 pmol/L) and better glycemic control (HbA1c≤ 7.0%/53.0 mmol/mol) had lower glucose uptake of T cells (**Figure 2**). Subsequently, the correlation analysis confirmed that the glucose uptake of T cells was negatively correlated with FCP and 2h-CP, and positively correlated with HbA1c, as the correlation of these clinical indications with 2NBDG^+^ and GLUT1^+^ in CD4^+^/CD8^+^ T cells was consistent (**Table 2**). Similar results were found in regression analysis (**Figure 3**). There was no difference in T cell glucose uptake in the subgroup with other clinical indicators such as the age of onset, gender, BMI, the status of islet autoantibodies, etc. (**Supplement Table 1**). The correlation analysis of T cell fatty acid uptake (%BODIPY and CPT1A of T cells) with clinical characteristics in T1D was not statistically different as well (**Supplement Table 2**).

**Table 2:** Correlation analysis between T cell glucose uptake and clinical indicators in T1D.

	T cell 2NBDG uptake				T cell GLUT1 expression			
	CD4^+^2NBDG^+^ (%)		CD8^+^2NBDG^+^ (%)		CD4^+^GLUT1^+^ (%)		CD8^+^GLUT1^+^ (%)	
	r	*P* value	r	*P* value	r	*P* value	r	*P* value
FCP	-0.316	0.003	-0.356	0.001	-0.266	0.015	-0.274	0.012
2h-CP	-0.278	0.010	-0.388	0.000	-0.233	0.035	-0.358	0.001
HbA1c	0.269	0.013	0.445	0.000	0.274	0.013	0.198	0.073

Spearman or Pearson analysis was used. FCP, Fasting C-peptide; 2h-CP, 2 hours postprandial C-peptide; HbA1c, hemoglobin A1c; 2NBDG, (2-[N-(7-nitrobenz-2-oxa-1, 3-diaxOL-4-yl) Amino]-2-deoxyglucose; GLUT1, glucose transporter 1.

### The Status of Glucose Uptake in T Cells Is Associated With Beta-Cell Function and Its Progression

In all T1D population, the median of %2NBDG of CD4^+^/CD8^+^ T cells and %GLUT1 of CD4^+^/CD8^+^ T cells were 5.81%, 5.82%, 4.88% and 3.72%, respectively. When the above 4 indicators of an individual were all higher than the median value, they were defined as the high glucose uptake group (n=37), and when they were all lower than the median value, they were defined as the low glucose uptake group (n=37). The other 12 cases were excluded from further analysis because their percentage of 2NBDG^+^ and GLUT1^+^ in CD4^+^/CD8^+^ T cells did not fulfill the above criteria (**Table 3**). The comparison of clinical features between the two groups showed that patients with higher T cell glucose uptake had a shorter diabetes course (1.4 months vs 5.9 months, *P*<0.001), lower FCP (121.8 pmol/L vs 211.3 pmol/L, *P*<0.001) and 2h-CP (271.8 pmol/L vs 702.4 pmol/L, *P*<0.001), and expected higher HbA1c levels (9.8% vs 6.6%, or 83.6 mmol/mol vs 48.6mmol/mol, *P*<0.001). Moreover, there were still significant differences in FCP, 2h-CP, and HbA1c between the two groups after the course of disease was corrected (**Table 4**).

**Table 3:** Clinical characteristics in T1D patients with different status of glucose uptake in T cells.

	Patients with low glucose uptake in T cells (n = 37)		Patients with high glucose uptake in T cells (n = 37)	*P* value
Age (years)	14.5 (11.5, 20.4)		13.4 (9.7, 20.7)	0.427
Male (%)	54.1		56.8	0.815
Course (months)	5.9 (3.6, 8.5)		1.4 (0.6, 3.2)	<0.001
DK/DKA-onset (%)	94.3		86.5	0.264
Hight (cm)	158.1 ± 16.4		151.1 ± 18.3	0.085
Weight (kg)	48.5 ± 15.4		42.5 ± 14.6	0.091
BMI (kg/m2)	18.8 ± 3.2		18.1 ± 3.1	0.298
FCP (pmol/L)	211.3 (159.1, 324.0)		121.8 (72.3, 163.8)	<0.001
2h-CP (pmol/L)	702.4 (454.2, 883.1)		271.8 (167.5, 425.7)	<0.001
HbA1c (%)	6.6 (5.8, 7.6)		9.8 (7.9, 12.4)	<0.001
HbA1c (mmol/mol)	48.6 (39.9, 59.6)		83.6 (62.8, 112.0)	
Insulin dose (U/kg/day)	0.5 (0.3, 0.6)		0.5 (0.3, 0.7)	0.360
GADA titer	0.43 (0.15, 1.12)		0.23 (0.03, 0.92)	0.062
IA-2A titer	0.57 (0.01, 1.76)		0.71 (0.00, 1.67)	0.944
ZnT8A titer	0.10 (0.01, 0.19)		0.07 (0.00, 0.25)	0.800
Number of positive autoantibodies (%)
1	13.5		27.0
2	40.5		45. 9	0.165
3	45.9		27.0

Data are expressed when normally distributed as means ± SD and when abnormally distributed as median (25th, 75th percentiles). The t test, Mann-Whitney U test, and χ2 test were be used. T1D, type 1diabetes; BMI, Body mass index; DK/DKA, ketosis/ketoacidosis; FCP, Fasting C-peptide; 2h-CP, 2 hours postprandial C-peptide; HbA1c, hemoglobin A1c; GADA, Glutamic acid decarboxylase antibody; IA-2A, protein tyrosine phosphatase antibody; ZnT8A, Zin transporter 8 antibody.

**Table 4:** Clinical characteristics in T1D after adjustment for disease course.

	Low glucose uptake		High glucose uptake		*P* value
	Mean ± Std. Error	95% CI	Mean ± Std. Error	95% CI	
FCP (pmol/L)	225.4 ± 19.3	186.9-263.9	147.5 ± 19.3	109.0-185.9	0.010
2h-CP (pmol/L)	613.5 ± 46.6	520.6-706.4	378.0 ± 46.6	285.1-470.9	0.002
HbA1c (%)	7.5 ± 0.4	6.7-8.4	9.8 ± 0.4	8.9-10.6	0.001

The ANCOVA analysis was used. Covariates appearing in the model are evaluated at the following values: course of disease= 4.43 months. FCP, Fasting C-peptide; 2h-CP, 2 hours postprandial C-peptide; HbA1c, hemoglobin A1; CI, Confidence Interval.

**Figure 4:**
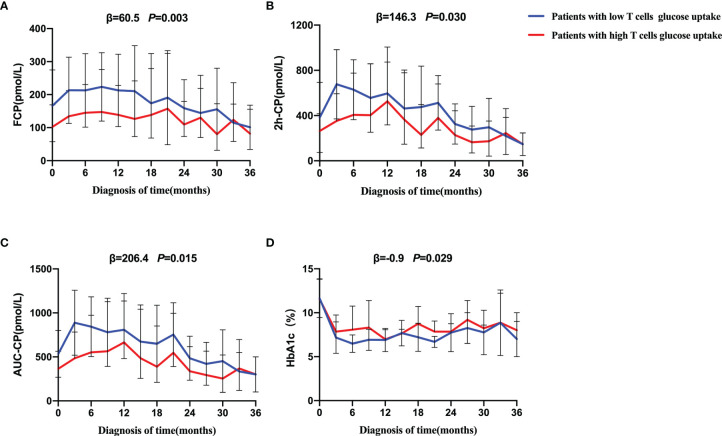
Progression of beta-cell function and trajectory of glycemic control in T1D during the follow-up. Generalized estimated equation (GEE) analysis was used to compare the dynamic tendency of FCP **(A)**, 2h-CP **(B)**, AUC-CP **(C)** and HbA1c **(D)** levels between the two groups of patients during a 36-month follow-up period. The blue line = patients with low T cells glucose uptake, red line = patients with high T cells glucose uptake. The error bar means SD. The statistical difference between the curves of the two groups was represented by *P* value. The low glucose group was on average *β* higher than that of high glucose group over a 36-month period. FCP, Fasting C-peptide; 2h-CP, 2 hours postprandial C-peptide; AUC-CP, Area under curve of C-peptide; HbA1c, hemoglobin A1c.

**Figure 5:**
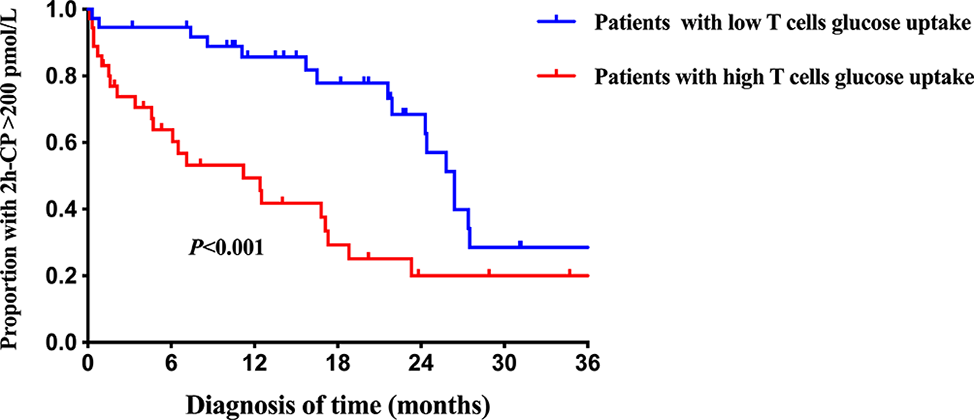
Survival curves of beta-cell preservation in T1D patients with different intracellular T cell glucose uptake status. The blue line = patients with low T cells glucose uptake, red line = patients with high T cells glucose uptake. 2h-CP>200 pmol/L as defined as beta-cells preservation. Log-rank survival analysis is used. 2h-CP, 2 hours postprandial C-peptide.

Further results on the dynamic changes in beta-cell function and HbA1c levels between the two groups were analyzed with follow-up data. It is noteworthy that the advantage of preserved beta-cell function and trajectory of glycemic control in patients with low T cells glucose uptake was maintained during the 36-month follow-up period (**Figure 4**). Compared with the high glucose uptake group, FCP, 2h-CP, and AUC-CP of the low glucose uptake group were 60.5 (95%CI: 21.1-99.8) pmol/L, 146.3 (95%CI: 14.1-278.5) pmol/L, and 206.4 (95%CI: 39.9-372.8) pmol/L higher, respectively, while HbA1c was 0.9 (95%CI: 0.09-1.74) % lower. In addition, this advantage of better beta-cell function in the low glucose uptake group persisted after adjustment for the course of the disease, C-peptide at onset, and HbA1c (**Table 5**). Furthermore, The survival analysis showed that the proportion of beta-cell preservation (defined as 2h-CP>200 pmol/L) in patients with low glucose uptake was also significantly higher than that in the high glucose uptake subgroup within 36 months after onset (**Figure 5**).

**Table 5:** Progression of beta-cell function in patients with different status of glucose uptake in T cells.

	FCP (pmol/L)		2h-CP (pmol/L)		AUC-CP (pmol/L)	
	β (95%CI)	*P*	β (95%CI)	*P*	β (95%CI)	*P*
**Model 1**						
Low glucose uptake	60.5 (21.1-99.8)	0.001	146.3 (14.1-278.5)	0.010	206.4 (39.9-372.8)	0.001
High glucose uptake	Ref		Ref		Ref	
**Model 2**						
Low glucose uptake	53.8 (20.2-93.4)	0.008	138.1 (10.2-299.1)	0.034	191.4 (29.1-353.7)	0.021
High glucose uptake	Ref		Ref		Ref	

FCP, Fasting C-peptide; 2h-CP, 2 hours postprandial C-peptide; AUC-CP, Area under curve of C-peptide; Ref, reference.

Model 1: unadjusted.

Model 2: adjusted for course of disease, C-peptide at onset, and hemoglobin A1c.

## Discussion

In this study, we showed that intracellular glucose uptake of CD4^+^/CD8^+^ T cells is abnormally enhanced in T1D and the status of glucose uptake in T cells is associated with beta-cell function and progression. To the best of our knowledge, this is the first study focusing on the association of T cell glucose uptake with clinical indicators and disease progression in T1D, providing new insights into the mechanism and intervention targets in the field of immunometabolism. It is well recognized that metabolism and the immunological state are inextricably linked (34). With the arising studies focusing on the intrinsic metabolic pathways of immune cells themselves such as glycolysis and Krebs cycle (35–37), immunometabolism has emerged as a major mechanism central to immune regulation. In this way, the metabolic signaling of immune cells drives cell fate. Considering the effect of the microenvironment on cell metabolism, immune cells can adopt programs specific for the tissues where they infiltrate and reside, highlighting opportunities of immunometabolism findings for clinical translation (38, 39).

However, puzzlingly, in the field of T1D where T cells are the primary mechanism, whether intra-T cellular metabolism is involved in the pathological process of T1D remains unclear. Through our T1D cohort study, we found the glucose uptake in T cells was significantly increased in T1D, which was mainly reflected as increased expression of GLUT1, a key transporter in the glucose metabolism pathway, and uptake of 2NBDG. Also, our results showed the abnormality of T cell glucose uptake was significantly correlated with islet function progression and glycemic control trajectory. Although no previous data has been reported about the association of intra-T cellular metabolism with beta-cell function progression, a few cross-sectional studies reported the status of T cell metabolism in T1D patients. Our results are similar to Kong BS’ research in 2021 which found that patients with T1D had enhanced CD4^+^ T cell glycolysis and decreased oxidative phosphorylation (40). Likewise, in NOD mice, increased 2NBDG uptake was also detected in NRP-V7-reactive T cells (a sort of diabetes pathogenic T cell) (21). A study by Vignali D found that GLUT1 was overexpressed in specific stem memory T cells by investigating the metabolic signature of naïve precursors stimulated with GAD65. And the similar metabolic signature was also found in influenza-specific T cells, indicating that this metabolic profile is not confined to autoreactive T cells (23). However, in this study, the 2NBDG uptake and mitochondrial mass and activity were similar among circulating CD8^+^ T cell subsets and no significant changes were found in T1D, indicating that the metabolic dysregulation of T1D not directly impact the basal metabolism of resting circulating T cell (23), which is not consistent with our results. It should be noted that the cells examined in the above studies were different. Upregulation of glucose metabolism was detected in pathogenic T cell populations with a specific antigen (NRP-V7 specific, GAD65, or influenza), but it is not uniformly in the circulating cells, which most will be of naïve phenotype, just as glucose metabolism is upregulated in CD4^+^ T cells (in the Kong BS study) but not in a subset of CD8^+^ T cells with different phenotypes (in the Vignali D study). We detected increased T cell glucose uptake in circulating CD4^+^ and CD8^+^ T cells from individuals with a large sample size with regular follow-up data, which might provide some solid information. Most importantly, our finding that abnormal glucose uptake of T cells is significantly correlated with beta-cell function and its progression. It offers potential markers for evaluating disease prognosis and suggests that the presence of a population of T cells characterized by elevated glucose metabolism enhances the immune attack on beta cells in T1D.

The causes of increased glucose uptake in T cells in T1D are unclear. Our primary consideration is hyperglycemia in the peripheral circulation of diabetes. However, there is little evidence of the relationship between hyperglycemia and the energy metabolism of immune cells. A study in a mouse model of autoimmune colitis with high glucose uptake found that hyperglycemia did not affect intracellular CD4^+^ T cell metabolism (41). However, it has also been reported that hyperglycemia can lead to increased glycolysis in activated T cells, resulting in increased IFN-γ production and non-antigen specific inflammation (42). Recent studies found T cell glucose metabolism decreased in patients with type 2 diabetes (33, 43) which may provide strong evidence that hyperglycemia is not the cause of changes in T cell glucose metabolism. In our study, the differential glucose uptake persists in beta-cell function progression after correction for glycation. Thus, the role of hyperglycemia may be limited. On the other hand, we consider that the enhanced glucose metabolism in T cells may be caused by susceptibility mediated by signaling pathways, oxidative stress, or immunomodulatory molecules. Enhanced T cell glucose metabolism has also been found in other autoimmune diseases including Systemic lupus erythematosus, Rheumatoid Arthritis, and Multiple Sclerosis (44–46). The metabolic abnormalities of T cells may be related to the production of autoantibodies to metabolic enzymes or the activation of the phosphoinositide 3-kinase (PI3K)-Akt and mammalian target of rapamycin (mTOR) pathway, which mediates susceptibility to immunometabolism abnormality in autoimmune diseases including T1D (16, 18). Oxidative stress and mitochondrial dysfunction have also been shown to play an important role in abnormal T cell metabolism in autoimmune diseases (47). In addition, the immune molecule programmed death 1 (PD-1) is a key regulator of T cell metabolic reprogramming, which has been found to be involved in the pathogenesis of T1D (30, 48, 49) and a study found that knocking out PD-1 on Treg cells in NOD mice significantly reduced the incidence of NOD mice by regulating intracellular metabolism (50).

Enhanced T cell metabolism was also detected in NOD mice and when prediabetic NOD mice were treated with 2-deoxyglucose to block aerobic glycolysis, islet antigen-specific T cell frequency and islet infiltrating lymphocytes decreased, beta-cell degranulation was improved (21). Similarly, in the adoptive transfer model of T1D, a competitive inhibitor of the glycolytic rate-limiting enzyme of CD4^+^ T cells reduced T cell responses to β cell antigen *in vitro*, thus reducing the immunopathological parameters associated with disease onset as 57% of animals remaining euglycemic at the end of the study period (22). Other studies also found that there are some potential immunotherapy drugs for T1D by inhibiting the T cell glucose metabolism pathway (51–53). These evidences, to some extent, suggest that T cell metabolism is an initiating factor in the pathogenesis of T1D. However, since the association between T cell metabolism and disease progression in T1D patients is poorly defined, our study provides human pathophysiological evidence for T cell glucose metabolism as a potential target.

Although the relationship between tissue infiltration and circulating immune cells is not fully understood, some evidence supports the hypothesis that effector cells migrate to the pancreas and that this autoimmune process persists in disease (54–56). The results of enhanced glucose metabolism in circulating T cells in T1D are consistent with the pathogenicity of T1D primarily caused by Th1 and Th17 and cytotoxic CD8^+^ T cells (54), as these cells require extensive glycolysis for energy during activation and differentiation. Furthermore, we found that changes in T cell glucose metabolism were correlated with beta-cell function progression within 36 months follow-up, suggesting that similar intracellular metabolic changes may also exist in T cells in islet infiltration. Anyway, further research will have to sort this out more completely.

Our study has some limitations, the most important of which is the failure to utilize the seahorse XF equipment to detection of intracellular glycolysis and aerobic phosphorylation pathways directly due to the limited number of cells. And the follow-up data on T cell metabolism is lacking to explore its mechanism during disease progression. Further, the intracellular glucose uptake is detected in bulk CD4^+^ and CD8^+^ T cells rather than pathogenic T cells with specific antigenic targets such as glucose-6-phosphatase catalytic subunit-related protein (IGRP) or GAD that can be detected by MHC tetramer technology as in other studies (21, 23), which is also one of our limitatons.

Overall, the association between intra-T cellular glucose uptake and progression of beta-cell function in T1D provides a promising possibility of T cell metabolism as biomarkers and potential targets. While there are trials focusing on immune intervention in T1D, the translation to specific T cell metabolism might provide a new and effective therapeutic strategy for T1D.

## Data Availability Statement

The original contributions presented in the study are included in the article/**Supplementary Material**. Further inquiries can be directed to the corresponding author.

## Ethics Statement

The studies involving human participants were reviewed and approved by The Second Xiangya Hospital of Central South University. Written informed consent to participate in this study was provided by the participants’ legal guardian/next of kin.

## Author Contributions

RT and TZ performed experiments and analyzed the data. RT wrote the manuscript. YX and XL reviewed and edited the manuscript. LF and JL contributed to the data collection and discussion. XL designed the study. All authors contributed to manuscript revision, read, and approved the submitted version.

## Funding

This work was supported by National Natural Science Foundation of China (Grant No 82070812), the science and technology innovation Program of Hunan Province (2020RC4044), and DMRFP_I_04 from SHMHDF.

## Conflict of Interest

The authors declare that the research was conducted in the absence of any commercial or financial relationships that could be construed as a potential conflict of interest.

## Publisher’s Note

All claims expressed in this article are solely those of the authors and do not necessarily represent those of their affiliated organizations, or those of the publisher, the editors and the reviewers. Any product that may be evaluated in this article, or claim that may be made by its manufacturer, is not guaranteed or endorsed by the publisher.
