# Supplementary material for: Enhanced T Cell Glucose Uptake Is Associated With Progression of Beta-Cell Function in Type 1 Diabetes
Source: Front Immunol. 2022 May 23;13:897047. doi: 10.3389/fimmu.2022.897047 (PMC9168918; doi:10.3389/fimmu.2022.897047)
Supplement: Supplementary file 1 [file DataSheet_1.docx]

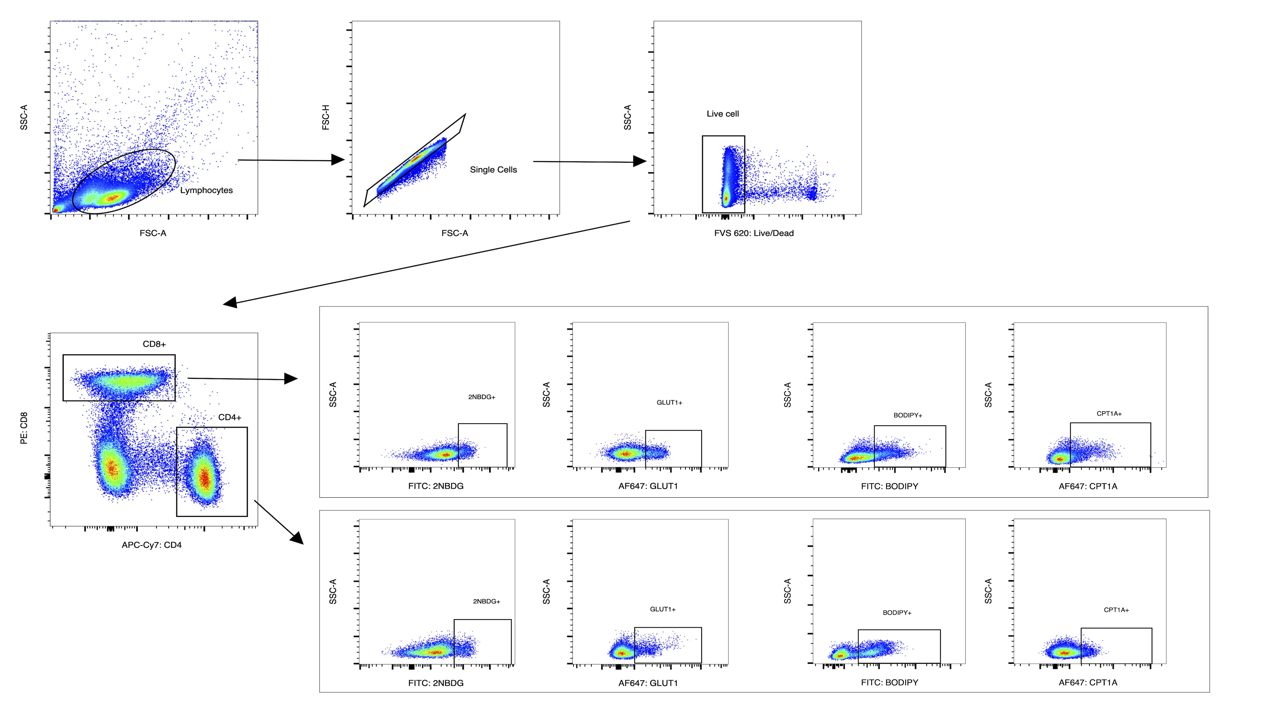


**Supplement Figure 1**  **Gating strategy for** **flow cytometry**

Cells were detected by the BD FACS Canto II system. The single fluorescence sample was used to calibrate the instrument regularly. Lymphocytes were circled by the forward/lateral scattering Angle (FSC-A/SSC-A), and 100,000 cells were collected inside the gate. Adhesion cells were removed by FSC-A/FSC-H. 2NDBG^+^, BODIPY^+^, GLUT1^+^, and CPT1A^+^ cells were marked.


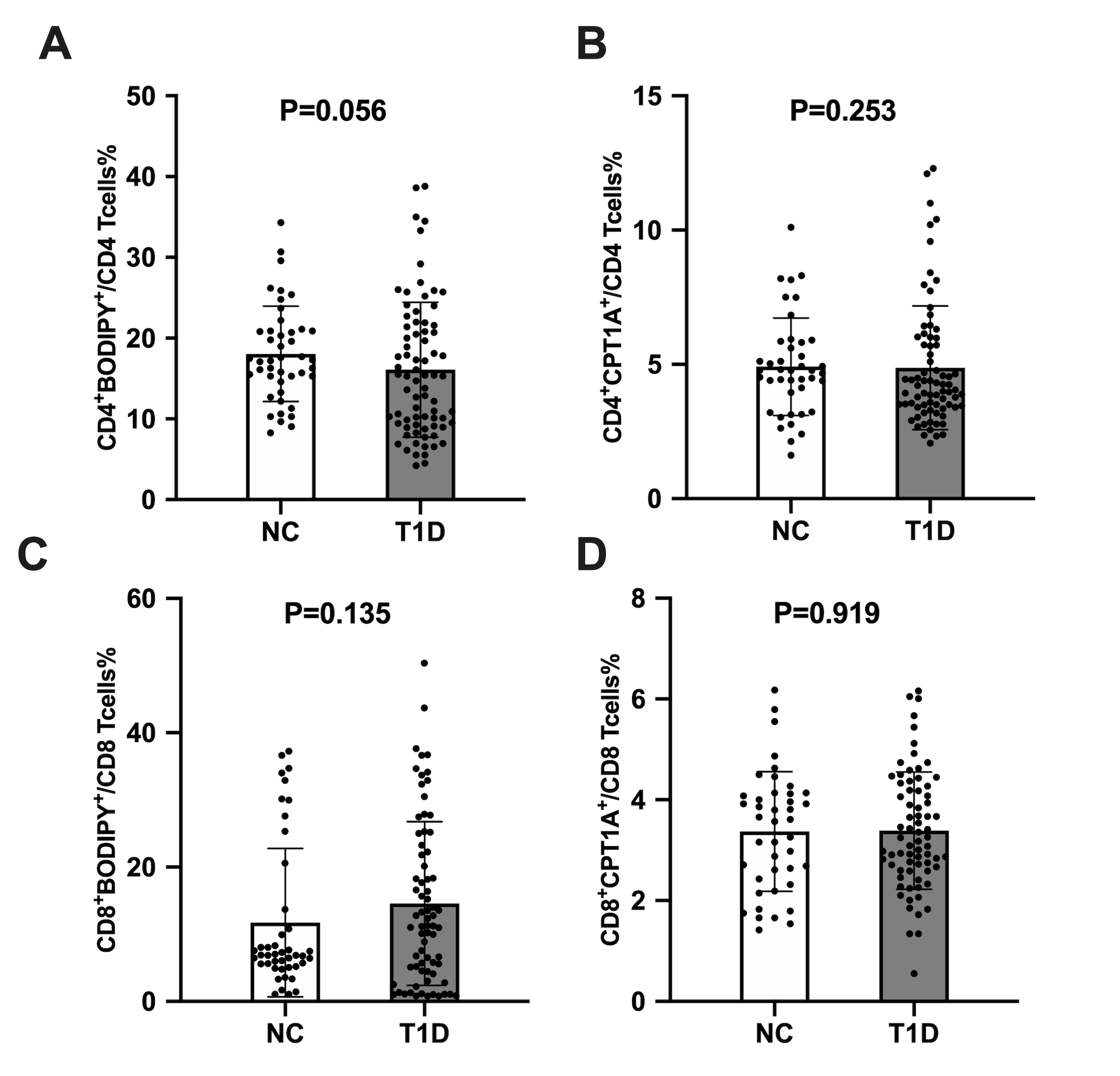


**Supplement Figure 2 BODIPY uptake and CPT1A expression of CD4^+^/CD8^+^ T cells**

There are no significant differences in CD4^+^ and CD8^+^ T cell fatty acid uptake between the T1D and NC. White squares = NC group, gray squares = T1D group. (A) The proportion of CD4^+^BODIPY^+^ cells in CD4^+^ cells. (B) The proportion of CD4^+^CPT1A^+^ cells in CD8^+^ cells. (C) The proportion of CD8^+^BODIPY^+^ cells in CD8^+^ cells. (D) The proportion of CD8^+^CPT1A^+^ cells in CD4^+^ cells. The bar and error bar = mean and SD. T1D, type 1diabetes; NC, normal control; CPT1A, carnitine palmitoyltransferase 1A.

**Supplement Table 1** **T cell glucose uptake in different T1D subgroups**

|  | | CD4^+^ T cells | | | | CD8^+^ T cells | | | |
| --- | --- | --- | --- | --- | --- | --- | --- | --- | --- |
|  |  | 2NBDG^+^ (%) | *P* value | GLUT1^+^ (%) | *P* value | 2NBDG^+^ (%) | *P* value | GLUT1^+^ (%) | *P* value |
| Sex | male | 6.31±2.76 | 0.484 | 6.48±2.93 | 0.121 | 5.48±2.69 | 0.702 | 5.38±3.72 | 0.504 |
|  | female | 5.88±2.35 |  | 5.70±2.86 |  | 5.05±1.98 |  | 4.87±3.24 |  |
| Age (years) | ≤18 | 6.18±2.37 | 0.429 | 6.14±2.80 | 0.996 | 5.32±2.63 | 0.654 | 4.51±2.68 | 0.116 |
|  | >18 | 5.96±3.12 |  | 6.13±3.23 |  | 5.25±1.77 |  | 6.90±4.76 |  |
| DK/DKA at onset | no | 6.79±2.29 | 0.368 | 6.12±1.97 | 0.764 | 5.87±1.90 | 0.250 | 6.36±4.26 | 0.366 |
|  | yes | 6.08±2.64 |  | 6.15±3.03 |  | 5.26±2.49 |  | 4.93±3.35 |  |
| BMI | <18.5 | 6.20±2.39 | 0.790 | 6.17±2.94 | 0.876 | 5.53±2.31 | 0.404 | 4.57±3.00 | 0.018 |
|  | 18.5-23.9 | 6.08±2.99 |  | 5.92±2.69 |  | 5.03±2.64 |  | 5.28±3.40 |  |
|  | >23.9 | 5.19±0.73 |  | 7.92±5.20 |  | 4.46±0.45 |  | 13.47±2.29 |  |
| HbA1c (%) | ≤7 | 5.37±2.76 | 0.003 | 4.91±2.13 | 0.001 | 4.38±1.74 | 0.005 | 3.90±2.66 | 0.002 |
|  | >7 | 6.65±2.34 |  | 7.00±3.09 |  | 5.93±2.62 |  | 6.06±3.79 |  |
| FCP (pmol/l) | ≤150 | 6.76±2.77 | 0.035 | 7.07±2.79 | 0.006 | 5.96±2.89 | 0.028 | 6.34±4.03 | 0.007 |
|  | >150 | 5.58±2.31 |  | 5.35±2.80 |  | 4.74±1.77 |  | 4.19±2.70 |  |
| 2h-CP (pmol/l) | ≤450 | 6.75±2.48 | 0.007 | 7.28±3.15 | 0.000 | 5.99±2.72 | 0.009 | 5.97±3.92 | 0.030 |
|  | >450 | 5.41±2.58 |  | 4.92±2.06 |  | 4.47±1.72 |  | 4.36±2.87 |  |
| Number of positive autoantibodies | 1 | 6.25±2.70 | 0.394 | 6.59±2.78 | 0.771 | 5.31±2.02 | 0.305 | 5.00±2.98 | 0.950 |
|  | 2 | 6.48±2.82 |  | 6.02±2.96 |  | 5.84±2.88 |  | 4.71±2.64 |  |
|  | 3 | 5.63±2.21 |  | 6.01±2.99 |  | 4.69±1.90 |  | 5.78±4.54 |  |
| GADA titer | <0.3 | 6.61±2.87 | 0.133 | 6.25±2.78 | 0.501 | 5.67±2.66 | 0.239 | 4.88±3.09 | 0.812 |
|  | ≥0.3 | 5.69±2.25 |  | 6.04±3.05 |  | 4.97±2.15 |  | 5.42±3.88 |  |
| IA-2A titer | <0.5 | 6.11±2.74 | 0.718 | 5.99±3.11 | 0.436 | 5.44±2.29 | 0.852 | 4.75±3.18 | 0.352 |
|  | ≥0.5 | 6.13±2.44 |  | 6.29±2.71 |  | 5.36±2.56 |  | 5.59±3.83 |  |
| ZnT8A titer | <0.07 | 6.11±2.66 | 0.919 | 6.34±3.25 | 0.702 | 5.05±1.96 | 0.840 | 4.83±3.14 | 0.503 |
|  | ≥0.07 | 6.20±2.54 |  | 5.97±2.59 |  | 5.58±2.78 |  | 5.54±3.87 |  |

Abbreviation: T1D, type 1diabetes; NC, normal control BMI, Body mass index; DK/DKA, ketosis/ketoacidosis; FCP, Fasting C-peptide; 2h-CP, 2 hours postprandial C-peptide; HbA1c, hemoglobin A1c; GADA, Glutamic acid decarboxylase antibody; IA-2A, protein tyrosine phosphatase antibody; ZnT8A, Zinc transporter 8 antibody.

**Supplement Table 2 Correlation analysis of fatty uptake with clinical features in T1D**

|  | CD4^+^ T cells | | | |  | CD8^+^ T cells | | | |
| --- | --- | --- | --- | --- | --- | --- | --- | --- | --- |
|  | % BODIPY | | % CPT1A | |  | % BODIPY | | % CPT1A | |
|  | *r* | *p* | *r* | *p* |  | *r* | *p* | *r* | *p* |
| Age(years) | -0.102 | 0.38 | -0.039 | 0.736 |  | -0.015 | 0.897 | -0.189 | 0.111 |
| Course of disease (months) | -0.017 | 0.884 | 0.138 | 0.235 |  | -0.116 | 0.330 | 0.040 | 0.741 |
| BMI(kg/m^2^) | -.086 | -.002 | -.083 | .021 |  | .460 | .984 | .483 | .859 |
| FCP (pmol/L) | -.118 | -.044 | -.082 | -.045 |  | .309 | .707 | .489 | .707 |
| 2h-CP (pmol/L) | .100 | -.113 | -.068 | -.163 |  | .394 | .333 | .571 | .174 |
| HbA1c (%) | -.080 | .155 | .053 | .128 |  | .493 | .185 | .657 | .288 |
| GADA titer | .087 | -.118 | -.082 | .026 |  | .453 | .310 | .492 | .829 |
| IA-2A titer | .124 | .212 | -.190 | .068 |  | .286 | .066 | .108 | .573 |
| ZnT8A titer | .020 | .015 | -.005 | .107 |  | .865 | .901 | .964 | .376 |
